# Curriculum and program evaluation in medical education: a short systematic literature review

**DOI:** 10.1097/MS9.0000000000002518

**Published:** 2024-08-30

**Authors:** Himayat Ullah, Sarwat Huma, Ghulam Yasin, Muhammad Ashraf, Qazi Tahir-ud-Din, Hossam Shabana, Junaid Sarfraz

**Affiliations:** a College of Medicine at Shaqra, Shaqra University, Shaqraa, Saudi Arabia; b Health Services Academy, Islamabad; c Faculty of Medicine, Department of Internal Medicine, Al-Azhar University, Cairo, Egypt; d Lady Reading Hospital, Peshawar, Pakistan

**Keywords:** curriculum evaluation, evaluation models, evaluation theories, program evaluation

## Abstract

Medical education is constantly evolving worldwide and facing various challenges. To cope with these, continuous and fruitful evaluation of an educational program is the need of the day. This study aims to know the purpose of evaluation, various theories related to program evaluation, and different models of curriculum and program evaluation. This will help educationists evaluate their programs fruitfully and effectively according to their needs and objectives. Different search engines including Medline’s PubMed interface, Google Scholar, and Cochrane Review databases using keywords, curriculum evaluation, evaluation models, and evaluation strategies in education, were searched without any date restrictions, and 20 full-text articles were selected for review and data extraction. While reviewing the literature it was found that most of the modern educational program and curriculum evaluation models are based on the reductionist, system, and complexity theories of evaluation. The experimental/quasi-experimental model is based majorly on the linear approach and reductionism, but its drawback is that it is impractical for the whole curriculum and sometimes ethically unfavorable. Kirkpatrick’s model, Philips’ model, the CIPP model, and the logic model are based on the system and complexity theory and are more practical in medical education. Each of these models has its advantages and limitations. In this review, the authors discussed the important distinctive features of these evaluation theories and models and their applicability and usefulness in evaluating different programs and curricula.

## Introduction

Highlights

- Curriculum is a dynamic process and behaves like a living thing, which is constantly under evolution.
- Curriculum and program evaluation is the backbone of modern education in general and medical education in specific.
- Various models have been proposed and practiced in the evaluation process in medical education, based on different theories of evaluation.

The term “curriculum” explicitly refers to a planned order of teaching or a perspective on student experiences to the instructional objectives of the teacher or institution. The planned interaction of students with instructional content, materials, resources, and mechanisms for assessing the achievement of educational goals may be included in a curriculum^1^.

The curriculum acts as a guide, a skeleton, or a blueprint for an educational program that provides a map towards the achievement of program goals and competencies. In other words, a program or more specifically an educational program is an ideology, while the curriculum is the practical framework for achieving that. A curriculum is a dynamic thing like a living thing. The curriculum or program developers can feel some relief once a curriculum is created, but their work is not finished. They would not know how successful their efforts are until the curriculum is reviewed and evaluated, not once, but rather periodically. It means that once designed, every curriculum should be evaluated from time to time for its growth and development, to assess its effectiveness, and to remove the flaws encountered during its delivery. It is accurate to argue that no curriculum is perfect because there are frequent elements that may have an impact on the curriculum that weren’t considered throughout its preparation. It is like a universal truth that every curriculum must be altered based on the findings of an evaluation if it is to be the most successful. To create a dynamic future in education, it must go on constructing and improving its current strengths. The pursuit of excellence is based on a distinct philosophy and purpose that necessitates well-constructed plans for improvement, incorporating novel modifications in educational practice, and routine evaluation of the program for the accomplishment of objectives and key components^2^.

Evaluation has been given many different definitions. According to one definition, it is simply the practice of determining the degree to which program objectives have been met by reviewing performance in different areas. As a result, evaluation is a decision-making process that involves judgment and is a comprehensive and ongoing investigation into the results of utilizing educational content and methodology to achieve certain objectives^3^.

Evaluation dates back to at least 1200 BC^4^. The idea of evaluating schools is not new in the United States; the first example of “evaluative standards” was established for the country’s secondary schools at the end of the 19th century^5^. But lately, there seems to have been a noticeable surge in interest in curriculum review in particular.

Curriculum evaluation is a very difficult, delicate, and uphill task that needs a lot of expertise. There are different theories, methods, and techniques of curriculum evaluation. The contemporary interest in theories and techniques of curriculum evaluation has been influenced by the public’s need for educational accountability, the experts’ calls for educational change, and the educators’ concurrent need for proof of results.

Unfortunately, a significant portion of this curiosity appears to have led to an unhealthy preoccupation with the evaluation process and results. It appears that a broader viewpoint and more varied techniques are required for a fruitful program evaluation.

Curriculum evaluation involves the methods of calculating and evaluating the degree to which the intended courses, programs, learning opportunities, and other elements as stated in the formal curriculum truly achieve the desired effects. Decisions on advancements and the future can be made if this procedure is carried out well.

The objective of this review is to discuss the purpose of evaluation, its types, different theories of evaluation, and different models that are used in program and curriculum evaluation.

## Search methodology

### Literature resources

Using a combination of keywords curriculum evaluation, evaluation models, evaluation strategies in education, and other free text terms deemed appropriate for the purpose, literature searches were conducted. No date limits were applied since the aim was to search all the available literature on medical education curriculum and program evaluation. A thorough search technique was created to go through Medline’s PubMed interface, Google Scholar, and Cochrane Review databases, with Medline as the main resource. The inclusion criteria were the original research, review articles (systematic and meta-analysis), and books/book chapters on the subject of curriculum and program evaluation in medical education. The editorials, commentaries, and articles not focusing specifically on education were excluded from the review.

### Data extraction and analysis

Using the PRISMA checklist, a total of 15 142 articles were found in the first search, the titles of which were reviewed, and 61 titles were found to be related to our topic of interest. The abstracts of these articles were reviewed by four different reviewers (H.U.; S.H.; G.Y. and M.A.). They enumerated the study characteristics and reported study findings on a preset criterion. Any disagreement was resolved by mutual discussion and consensus. The quality of the research was assessed by applying Best Evidence Medical Education (BEME) quality criteria by Buckley *et al.* ^6^. From the final full-text publications of 20 articles, we took into account the research’s heterogeneity and extracted the different characteristics of theories and models underlying educational evaluation programs. The PRISMA Flowchart below shows the search process.

## Results

The studies reviewed are summarized in Table 1 below.

**Table 1:** List of studies reviewed.

No.	Study	Type	Year	Subject
1	ACGME^7^	Webpage	Created 2010, Accessed 2023	Purpose of evaluation
2	Goldie^8^	Journal Article	2006	Objectives of evaluation
3	Thomas *et al.* ^9^	Journal Article	2000	Objectives of evaluation
4	Gilroy *et al.* ^10^	Journal Article	2001	Objectives and theories of evaluation
5	UNFPA^11^	Webpage	Created 2001, Accessed 2023	Objectives of evaluation
6	Patten *et al.* ^12^	Book	2010	Objectives of evaluation
7	Stufflebeam *et al.* ^13^	Book	2014	Objectives of evaluation, theories and models
8	Mennin *et al.* ^14^	Journal Article	2010	Reductionism
9	Geyer *et al.* ^15^	Book	2005	Reductionism and complexity theory
10	Durning *et al.* ^2^	Journal Article	2007	Reductionism
11	Von *et al.* ^16^	Journal Article	1972	System Theory
12	Cambell *et al.* ^17^	Book	2015	Experimental/Quasi-experimental Model
13	Suchman *et al.* ^18^	Journal Article (Document)	1965	Experimental/Quasi-experimental Model
14	Stufflebeam *et al.* ^19^	Book	2000	CIPP Model
15	Frechtling *et al.* ^20^	Book	2007	Logic Model
16	Kirkpatrick *et al.* ^21^	Book	2006	Kirkpatrick’s Model
17	Arthur *et al.* ^22^	Journal Article	2003	Kirkpatrick’s and Philips’ Model
18	Bewley *et al.* ^23^	Journal Article	2013	Kirkpatrick’s Model
19	Hamemoradi *et al.* ^24^	Journal Article	2014	Evaluation models
20	Bisgaard *et al.* ^25^	Journal Article	2018	Philips’ Model

### Objectives of evaluation

While reviewing the literature we found that curriculum evaluation has two main purposes,

1. Whether it is accomplishing its goals
2. Assessing the program’s quality

Broadly speaking, the curriculum is evaluated by the educationists for internal and external factors. Primary external factors can frequently be found in financial sources that encourage educational innovation, requirements of medical education accreditation organizations, and other groups or people to whom educators are accountable^7^. While enabling educators to learn essential information about their programs and maintain ongoing program development, an effective program assessment procedure promotes accountability^8^.

The evaluation of the quality and suitability of any program for a particular purpose is of utmost importance, hence determining the program’s level of quality against specific benchmarks and key performance indicators (KPIs) is a key factor in curriculum evaluation^9,10^. The evaluator or reviewer of any curriculum evaluation typically has a list of questions in mind that they hope to address through their work. How equipped are graduates to enter the workforce in healthcare facilities, for instance? or What instructional techniques are employed to make sure that the graduate is capable of making wise clinical decisions? etc. Usually, curriculum modifications are decided upon based on the replies to these questions^11^.

Such a wide spectrum of requirements has not always been accommodated by a single evaluation approach. For many years, evaluation specialists concentrated only on gauging program results^12^. Although these are still trusted as a part of evaluation models, a greater emphasis on program improvement is made possible by the support provided by more recent evaluation methods/models, that follow a more holistic approach, for better learning about the dynamic processes within the educational programs^12,13^. The older quasi-experimental evaluation model will be discussed first, followed by a description of some of the theoretical concepts that have influenced both older and modern evaluation techniques. Finally, we will discuss some of the newer, more potent models that are based on more recent theories.

### Theories of curriculum evaluation

#### Reductionist theory

Many of the educational evaluation techniques started evolving when the concept of the divine intervention changed to experimentation and research^14^.

Reductionism means dividing a whole into its different constituents and then studying and evaluating the effect of each constituent separately. In medical education, evaluating different components of a curriculum can give an idea of the outcome beforehand. Reductionist or linear thinking believes that program success or failure to achieve desired results can be explained once the components influencing the outcome are identified and thus managed. This was predicated on the idea of order which anticipates that with the growth of civilization and knowledge, there would be a transition from disorder to order. A phenomenon can be reduced to and comprehended in terms of its constituent pieces by dissecting it. Because order is the norm, outcomes could be expected with some accuracy, and processes could be controlled or predicted because they would follow clear and organized pathways^15^.

This theory is the basis of several evaluation techniques and models used in education for the past one century, for example Logic Model and the Before, During, and After model^2^.

#### System theory

This theory states that an educational program or a curriculum behaves like a social system, made up of individual components that interact and are interrelated with one another while living in and engaging with the program’s environment so that the sum of the individual component is not mathematically equal to the result or outcome of that program or curriculum.

The concept of system theory can be traced back to the work of Bertalanffy, a biologist who proposed that. He states that the organization of a living thing is its fundamental characteristic; the conventional study of its parts and processes cannot give us a comprehensive explanation of the vital phenomena; nor can it provide us information regarding the coordination of its parts and processes^16^. So according to system theory, the outcome of a curriculum or a program is not merely the sum of its constituent parts but rather its more complicated interaction between the components of that program so that its outcome cannot be simply explained by the summation of the components (as opposed to the reductionist approach).

#### Complexity theory

The concept of complexity theory is based on the extrapolation of the system theory. The educational program or curriculum is a complex interplay of its different constituents. It is an open system in which changes happen constantly and inevitably, so that one cannot predict the exact outcome beforehand. The characteristics of program participants, the influence of stakeholders or regulators, the dynamic nature of the knowledge underpinning a discipline, professional practice patterns, and the environment in which the educational program operates are just a few of the many internal and external factors that impact medical education programs^15^. Because medical education programs consist of various components that interact with one another, it is helpful to think of them as complex systems. The goal of complexity research and theory is to accept the broad range and depth of systems where uncertainty and unpredictability are inherent. The complexity theory is the key to the evaluation of a curriculum or a program in modern medical education.

### Curriculum evaluation models

#### The experimental/quasi-experimental models

It is one of the oldest and earliest evaluation models influenced by the reductionist approach. This model adheres to the traditional reductionist theory as it analyses and evaluates a program by linearly correlating its components to predict the outcomes^17^. This model can be used in a fully controlled experimental environment but is less effective in educational programs as experimental and controlled environments cannot be achieved practically, thus affecting the validity and accuracy of the evaluation^18^.

**Figure 1:**
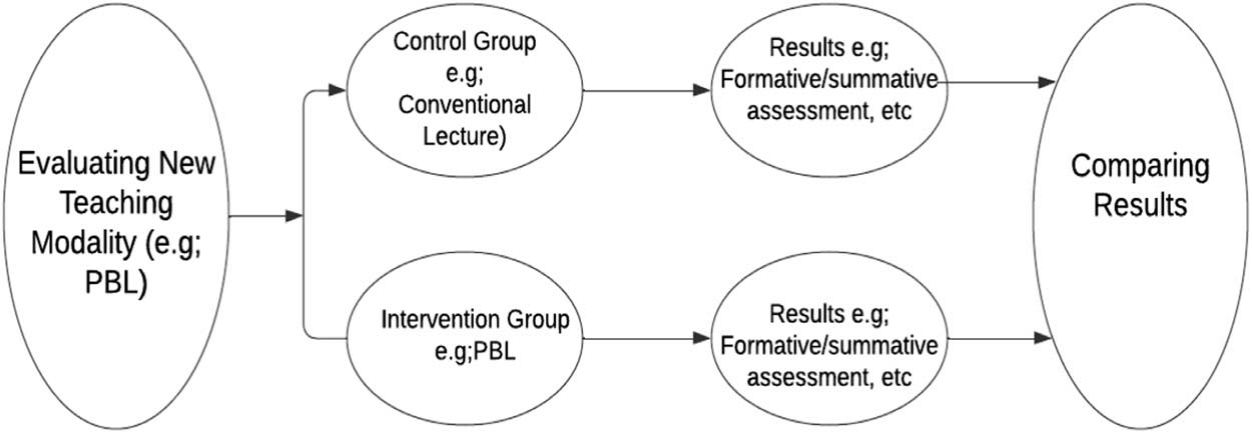
The experimental/quasi-experimental model. PBL, problem based learning.

Practically, this model evaluates the effectiveness of new teaching methods, curricula, or educational tools by making a control group and an intervention group and then comparing their results, for example comparing the results of two groups, one taught by problem-based learning and the other by conventional lecture. The advantage of this model is that it is highly valid and standardized, but it’s not practical for the whole curriculum and also not ethically appropriate by withholding a superior mode of learning from a group of students. Figure 1 shows the schema of the experimental/quasi-experimental model.

#### The CIPP model

The CIPP model of evaluation is among the most useful program evaluation models and focuses on all the components of an educational program, which are Context, Input, Process, and Products^13,19^. The CIPP model addresses all the phases of the educational program from planning to implementation to assessment. The four components of the CIPP evaluation model are;

1. *Context*: Context evaluation means, what has to be done in a specific program. What are the specific needs of the program, what problems can come across, what are the available resources and what are the required resources?
2. *Input*: Input evaluation means, how the program should be run. What are the possible strategies to fulfill those specific needs of a program? how practical are these strategies, any alternative to these strategies, and what is their economic feasibility.
3. *Process*: Process evaluation means how the program’s execution is done as compared to the plan, and how the different components and activities are timed, conducted, budgeted, and documented. Any issues in the above-mentioned points, the reasons for these issues and the way out adopted should be evaluated. The stakeholders’ satisfaction should also be considered and evaluated.
4. *Product*: Product evaluation means how successful the program was. It evaluates the outcomes of the program or curriculum. Product evaluation is divided into evaluations of impact, effectiveness, sustainability, and transportability;
  - Impact evaluation measures how well a program reaches its intended audience.
  - Effectiveness rates the importance and caliber of results.
  - Sustainability measures how well a program’s contributions are institutionalized and sustained over time.
  - Transportability evaluation determines how well it could be modified and implemented in another similar setting.

Taking an example, suppose we are evaluating the need for a clinical skills course for the preclinical years’ students in order to cope up with the demands of modern medical education. This is context evaluation.

Next, we analyze how the curriculum for this course should be designed. There should be clinical skill labs with different types of mannequins and the involvement of clinical faculties and hospital personnel. This is an input evaluation.

After this, we shall evaluate this process by auditing the skill lab sessions by different sources, including students and faculty’s feedback as well 360 feedback. This is process evaluation.

The final step is the assessment of the students against a set benchmark, by formative and summative assessment and other key performance indicators (KPIs).

**Figure 2:**
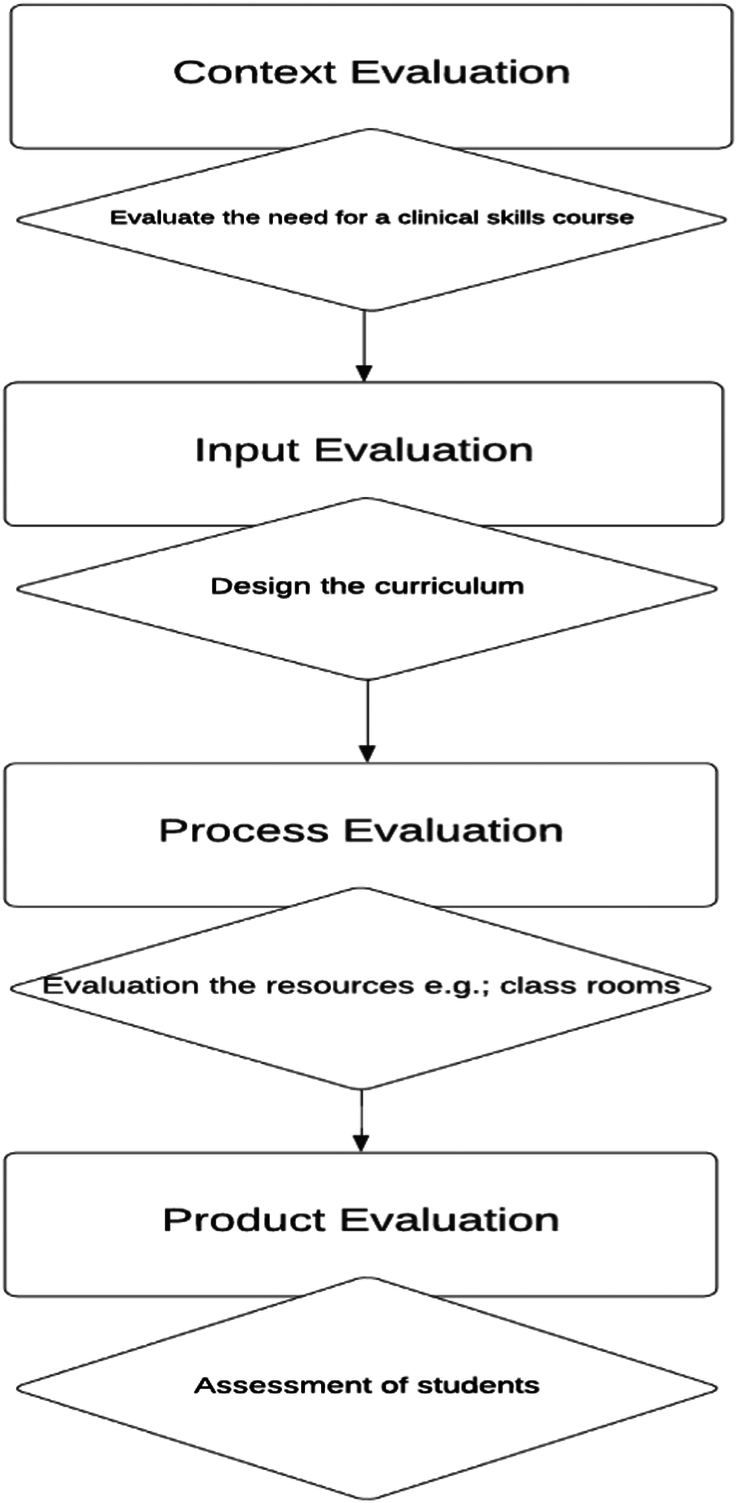
The CIPP model.

The advantage of this model is that it provides a thorough evaluation of the whole program, involving all the stakeholders, is widely applicable, and flexible. The only challenging aspect of this model is resource intensiveness, expertise dependent, and extensive data-driven. Figure 2 shows the schema of CIPP model.

#### The logic model

The logic model utilizes the concept of system theory^20^. This model explains the rational connections between the resources, actions, audiences, outputs, and program outcomes concerning a particular issue or circumstance. This model is mainly used in planning a program, so once it is described on the basis of the logic model the results and outcomes can be predicted and explained. The four main components of this model are;

*Inputs*: Inputs means the resources of the program, including the learning resources and the infrastructure.

*Activities*: Activities means the collection of intended “treatments,” “strategies,” “innovations,” or “changes” for the educational program. Generally speaking, activities are performed in the sequence given in the Model.

*Outputs*: Output means that the activities that are underway or completed, resulted in certain intended outputs or at least one output.

*Outcomes*: Outcomes means to what extent the intended objectives are achieved after the completion of the program. These outcomes may be short, intermediate, or long term.

Let’s take an example of a new clinical skill program for undergraduate medical students on the basis of the logic model.

In the first step, we define the resources required like tutors, clinical skill labs with mannequins, hospital wards, etc. These are inputs. The next step is defining the modes of information transfer, which may include workshops, simulations, tutorials, bedside teaching sessions, and multisource feedback. These are called activities. The third step is to evaluate the implementation and completion of these activities, like how many workshops, skill lab sessions, tutorials, and bedside teaching sessions have been conducted, how many students have participated in these, and what feedback from different sources has been received. This is output. In the final step, we evaluate the program against the objectives achieved (improvement in skills and knowledge) and competencies gained (improvement in patient care). This is called outcome.

**Figure 3:**
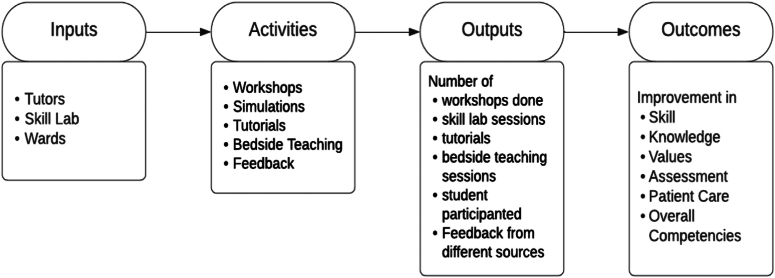
The Logic model.

The advantage of the logic model is its structural clarity and organization. The main challenge faced in the logic model is extensive planning which is quite resource-intensive, and its static nature, since medical education is quite dynamic and needs continuous modification based on the feedback during the program. Figure 3 shows the schema of the logic model.

#### Kirkpatrick’s four-level evaluation model

Kirkpatrick’s four-level model of learning evaluation was introduced in 1950, which is a comprehensive model of program evaluation^21^. This model was adapted by several medical educationists^22,23^. It has undergone multiple alterations since then, although the model’s primary ideas, or the four primary levels of evaluation, are still in place as of right now^24^. The four levels of the Kirkpatrick evaluation model are:

1. *Reaction:* It is the learners’ level of satisfaction regarding the program.
2. *Learning:* The level of learning during the program that is evaluated by assessing different learning domains (cognitive, psychomotor, and affective domains). It is evaluated during the program.
3. *Behavior:* Behavioral changes of the learners, that are evolved during the educational program in the context of the learning domains. It is evaluated at the end of the program.
4. *Result:* The final product of the program is a broader and longer view, like in real-life practices.

If we take an example of a new clinical skill program for undergraduate medical students, level 1 is to evaluate how students are satisfied with the clinical skill program and this can be done by taking feedback. The 2nd level is to evaluate the extent of skill and knowledge achieved by the students, done by pre-session and post-session evaluation. The 3rd level is to evaluate, to what extent students apply this skill in clinical settings that is while examining patients in the hospital. The 4th level is to evaluate the effects of the program on the students’ competencies that is improvement in the level of patient care.

**Figure 4:**
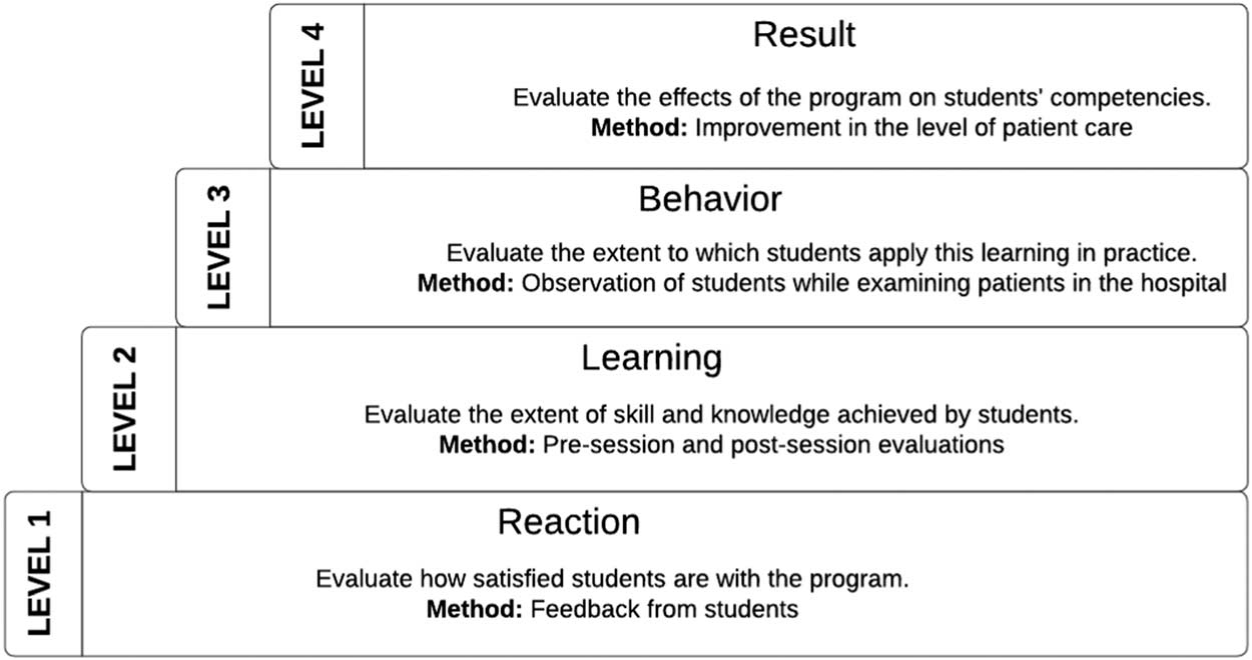
The Kirkpatrick’s model.

The main advantage of this model is that it is comprehensive and evaluates both the immediate as well as long-term effects of the program. The main challenge posed in this model is difficulty in evaluating the learners’ behavior in the long term, its duration, and resource intensiveness. Figure 4 is the schematic representation of the Kirkpatrick’s model.

#### Philips’ model:

Phillips’ model is basically the extension of Kirkpatrick’s model and is formulated by adding a fifth evaluation level to the four levels of Kirkpatrick’s model. This fifth component is the return on investment and is more related to the financial outcomes of the program^22,25^.

By taking the example of the new clinical skill program, the 5^th^ level that is return on investment would be early diagnosis and so decreased hospital stay, avoiding useless investigations, decreased patient readmissions, etc.

The extra advantage of Philips’ model over Kirkpatrick’s model is the economic aspects and cost-effectiveness of an educational program. The challenge that can be faced is the complexity of the model as calculating the cost can be difficult most of the time. Another issue is that this model can produce bias by withholding an educationally useful program on the basis of costs.

## Summary of conclusions

Educational programs and curricula need constant changes towards the betterment to cope with the widespread evolution in knowledge. Several approaches for evaluating programs and curricula have been put forth by academics and researchers in the subject. While some of these models have been well-examined, others have not. In a similar vein, some have consistently faced challenges and criticism. All of these evaluation models eventually seek to determine whether or not a curriculum or program has achieved its goals. These program evaluation models have changed enormously over time in various aspects including the concepts, the structure, the components and implementation strategies, and also the outcome measurements. The simple cause-and-effect link between program expectations and actual results is no longer the main focus of the study. Instead, they favor prioritizing ideas like competency, costs, effectiveness, and sufficiency^13,26^. As a result, the field of program and curriculum evaluation is becoming more complex^27^. Effective program evaluation is significantly hampered by the programs’ complexity, a fact best understood by educationists and curriculum developers. In conclusion, the selection of a particular evaluation model is contingent upon a number of factors that the assessor(s) must take into account beforehand. By taking into consideration the ideas that impacted the development of popular evaluation models, educationists and curriculum developers can obtain insight into various models of evaluation that can help accomplish their tasks and needs. The typical experimental and quasi-experimental evaluation methods, which are based on the rigid linearity of reductionist theory, may be too restrictive to account for the recognized complexity of educational programs. Likewise, Kirkpatrick’s four-level model also assumes linear correlations between program components and the results, which may help assist evaluators in identifying meaningful learner outcomes. Unlike previous evaluation models, the Logic Model is more inclusive because of its foundation in systems theory, which encourages evaluators to include the program’s environment in the evaluation process. Lastly, the CIPP model is congruent with system theory and, to a lesser extent, with complexity theory, it is adaptable enough to include studies that support ongoing program improvement as well as summative studies of completed program outcomes.

## Future trends in medical education program evaluation

After the beginning of 21^st^ century, there is a blast of evolution in medical education. The various models of curriculum and program evaluation are becoming more and more complex, and multiple evaluation methods and parameters are becoming part of these model. These parameters include but are not limited to, competencies bases evaluation, evaluating portfolios, technology-based evaluation, interprofessional education evaluation frameworks, community-based evaluation of the program, evaluation of health professionals’ mental and social health, and artificial intelligence (AI) based evaluation of the curriculum.

## Ethical approval

Ethics approval was not required for this review.

## Consent

Informed consent was not required for this review.

## Source of funding

Not applicable.

## Author contribution

All authors contributed equally to this paper and equally approved the submission. H.U., S.H. and J.S.: conceptualization and writing of initial draft; G.Y. and M.A: writing, editing, and revision of drafts; H.S. and Q.T.: visualization and writing.

## Conflicts of interest disclosure

The authors have no conflicts to disclose.

## Research registration unique identifying number (UIN)

Not applicable.

## Guarantor

Not applicable.

## Data availability statement

Not applicable.

## Provenance and peer review

Not applicable.
